# Evaluation of primary physicians’ awareness levels of non-ossifying fibromas and fibrous cortical defects by using referral documents

**DOI:** 10.1097/MD.0000000000041754

**Published:** 2025-04-04

**Authors:** Manabu Hoshi, Yoshitaka Ban, Naoto Oebisu, Tadashi Iwai, Naoki Takada, Masanari Aono

**Affiliations:** a Department of Orthopedic Surgery, Osaka City General Hospital, 2-13-22 Miyakojima-honor, Miyakojima-Ku, Osaka, Japan; b Department of Orthopedic Surgery, Osaka Metropolitan University Graduate School of Medicine, 1-4-3 Asahi-Machi, Abeno-Ku, Osaka, Japan.

**Keywords:** awareness, FCD, NOF, primary physician, referral document

## Abstract

This study aimed to investigate the awareness levels of primary physicians regarding non-ossifying fibromas (NOFs)/fibrous cortical defects (FCDs) using patient referral documents. Referral documents of 62 male and 46 female patients (mean age: 11 [5–32] years) who were diagnosed with NOFs/FCDs based on radiological findings were retrospectively reviewed. The suspected diagnoses by primary physicians were classified as “NOF/FCD,” “Benign,” “Intermediate,” “Malignant,” and “No description.” The Ritschl radiological classification of NOFs/FCDs and the relationship between the stages and suspected diagnoses were examined. NOFs/FCDs were diagnosed in 40 patients. The classifications were “Benign,” “Intermediate,” “Malignant,” and “No description’’ in 12, 3, 5, and 48 patients. Osteosarcoma was suspected in 4 patients. Among 63 patients with stage A Ritschl classification, NOFs/FCDs were diagnosed in 24 and osteosarcoma was suspected in 3. In 30 patients with stage B, NOFs/FCDs were diagnosed in 11 and osteosarcoma was suspected in one. Among patients with stages C and D, 3/10 and 2/5 patients, respectively, were diagnosed with NOFs/FCDs and a malignant bone tumor was suspected in 1 case with stage D. The suspected diagnosis was ``No description’’ in 46%, 43.3%, 40%, and 40% of patients with stages A, B, C, and D, respectively. The ``primary physicians” awareness levels of NOF/FCD seemed insufficient, owing to a high rate of “No description.” Plain radiography is a diagnostic technique for NOFs/FCDs, and NOF/FCD lesions commonly develop in children and young adults. Primary physicians need to be educated about the typical NOF/FCD radiological findings.

## 1. Introduction

Non-ossifying fibromas (NOFs) and fibrous cortical defects (FCDs) are tumor-like lesions that develop in the metaphyses of long bones, such as the femur, tibia, and fibula. These types of lesions are common, particularly in children and young adults. Plain radiography is usually used for diagnosing NOFs/FCDs, which are characterized by elliptical, well-demarcated radiolucent, eccentric, bony lytic lesions mainly confined to the cortex in the metaphyseal portions of long bones, especially the distal femur, proximal or distal tibia, and proximal humerus.^[1]^ NOFs/FCDs are common in daily orthopedic practice, with an estimated incidence of 30% to 40%.^[2]^

The prevalence of NOFs and FCDs is reported to be 2.3% and 7.0%, respectively, among lesions developing around the knee in children and young adults.^[3]^ While most patients with NOFs/FCDs initially visit an orthopedic surgeon, some may first consult other primary care physicians before being referred to orthopedic oncologists. Despite their high incidence,^[2]^ primary physicians often lack high familiarity with NOFs/FCDs. Some misdiagnose these lesions as malignant tumors, such as osteosarcoma, due to similar age groups and clinical presentations.^[4]^ This study retrospectively assessed primary physicians’ awareness of NOFs/FCDs by reviewing referral documents.

## 2. Materials and methods

### 2.1. Study design and patient selection

Data from an institutional database was reviewer, identifying 116 patients referred and diagnosed with NOFs/FCDs between October 2006 and June 2024. Diagnosed was confirmed based radiographic evidence of well-circumscribed radiolucent lesions in the metaphyses of the long bones. FCDs are confined to the cortices of the long bones, whereas NOFs might extend into the bone marrow. In this study, NOFs/FCDs was unified according to the World Health Organization’s classification of tumors.^[2]^ In case the lesion size was relatively large and the radiological findings were not typical of NOFs/FCDs, magnetic resonance imaging (MRI) was performed to confirm the diagnosis. The images were reviewed and confirmed by 3 orthopedic oncologists certified by the Japanese Orthopedic Association. Eight cases were excluded owing to inconsistencies. Moreover, confusing lesions, such as osteoid osteomas, osteofibrous dysplasia, and probable secondary aneurysmal bone cysts, were excluded from this study. Patient characteristics including age, sex, stage, initial symptoms, affected site, number of lesions, laterality, affiliated institution, and specialty of the primary physicians were reviewed (Table 1).

**Table 1:** Demographic data of the patients with non-ossifying fibroma and fibrous cortical defect.

Factors		Number
Age	Median (range)	11 y.o. (5–32)
Sex	Male	62
	Female	46
Symptom	Pain	69
	Incidental discovery	36
	Discomfort	1
	Numbness	1
	Pathological fracture	1
Affected site	Femur	67
	Tibia	32
	Fibula	7
	Humerus	2
Number of lesions	Single	107
	Multiple	1
Laterality	Lateral	98
	Bilateral	10
Affiliated institution of previous physicians	Hospital	45
	Clinic	63
Specialty of previous physicians	Orthopedic surgeon	105
	Pediatrician	2
	Nephrogist	1

### 2.2. Evaluation of primary physician’s awareness

**Figure 1.**
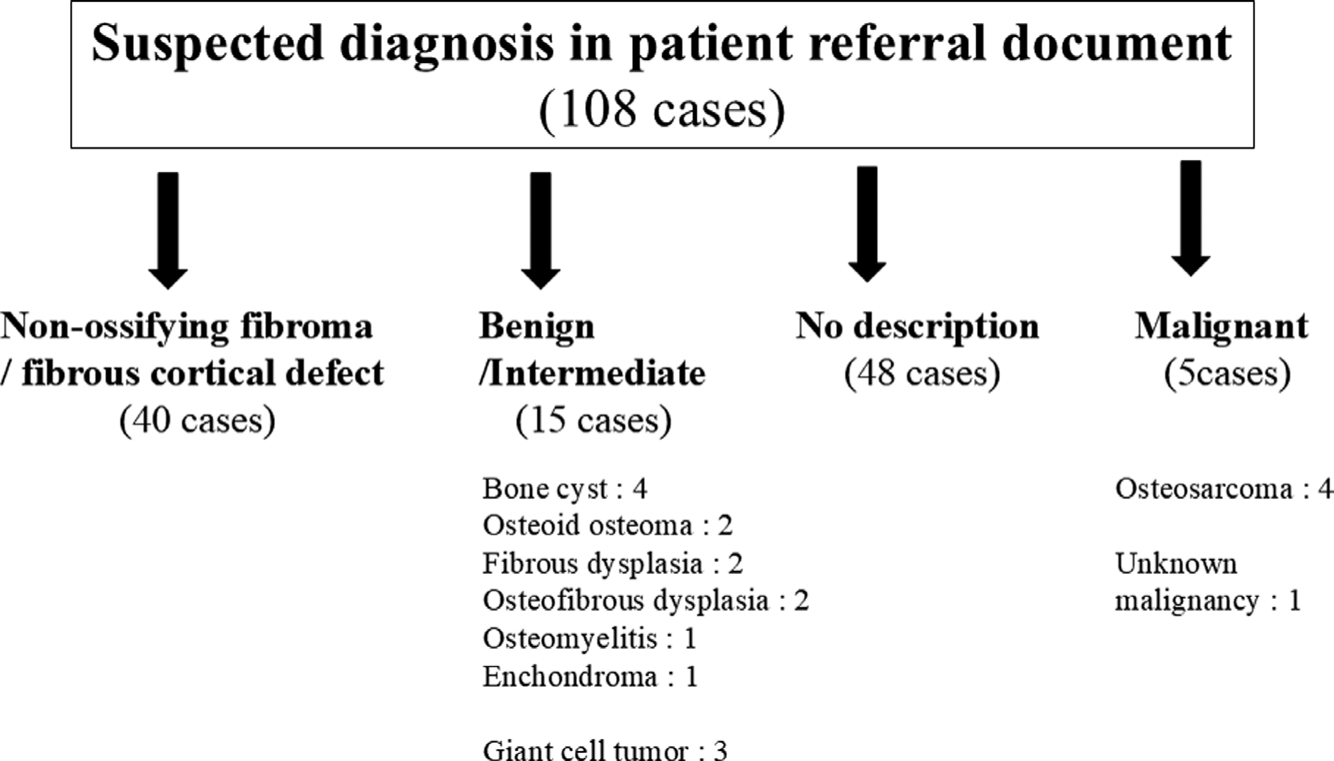
Chart of suspected diagnosis in patient referral document.

Patient referral documents were used to assess the presence or absence of NOFs/FCDs, and any other suspected diagnoses, to evaluate primary physicians’ awareness of NOFs and FCDs. The suspected diagnoses by primary physicians were classified as “NOF/FCD,” “Benign,” “Intermediate,” “Malignant,” or “No description” based on World Health Organization criteria (Fig. 1). Benign tumors, tumor-like lesions, and osteomyelitis were categorized as “Benign.” Giant cell tumor of bone was categorized as “Intermediate.” Osteosarcoma and probable malignancy (unknown bone tumor) were categorized as “Malignant.” No description of suspected diagnosis was categorized as “No description.”

### 2.3. Diagnostic process and radiological investigations

**Figure 2.**
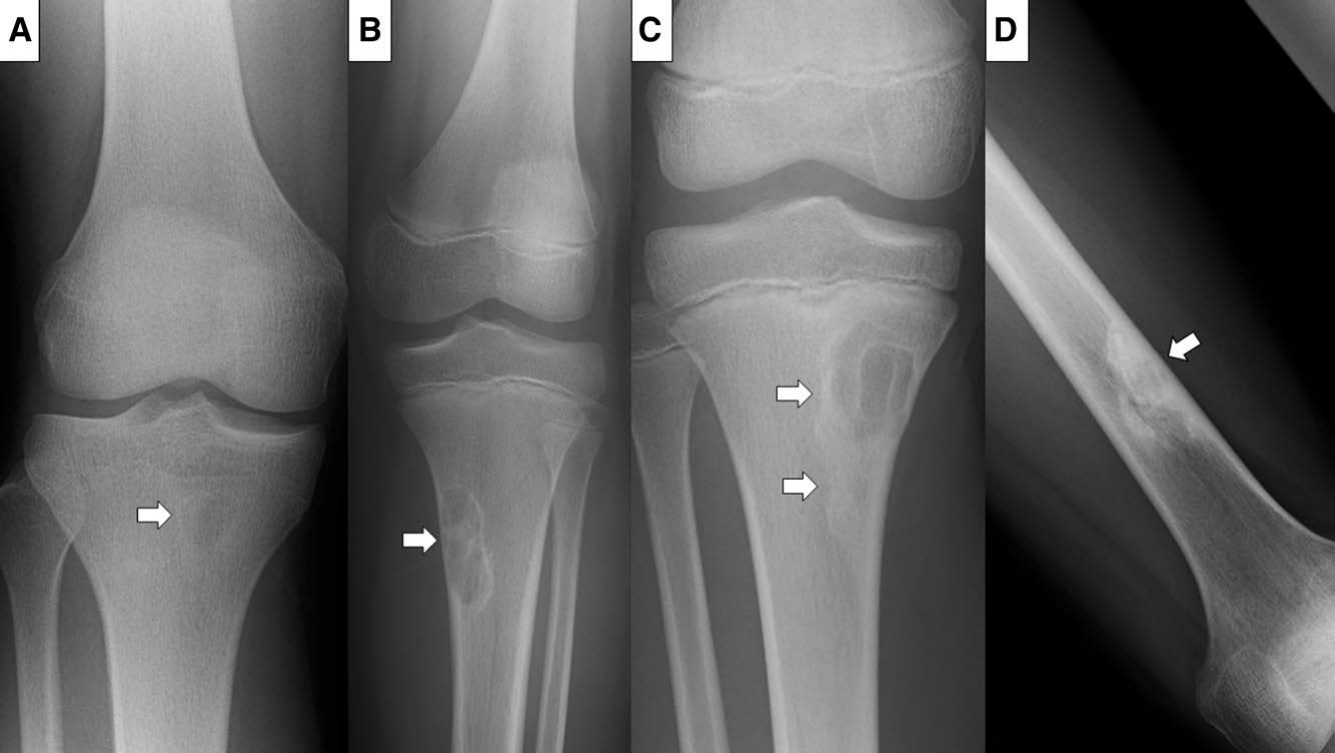
(A) Ritschl stage A. A 14-year-old male patient with a fibrous cortical defect involving the proximal tibia. The lesion was small and oval with a sclerotic border. (B) Ritschl stage B. An 11-year-old male patient with a non-ossifying fibroma involving the proximal tibia. The lesion was polycystic with a sclerotic border and thinning cortex. (C) Ritschl stage C. An 11-year-old male patient with a non-ossifying fibroma involving the proximal tibia. Typical sclerosis started from the distal side. (D) Ritschl stage D. A 25-year-old male patient with a non-ossifying fibroma involving the distal femur. Complete sclerosis was noted.

To determine the diagnostic process in the previous institution, that is, prior to referral to our hospital, the presence or absence of radiological information on plain radiography, MRI, computed tomography (CT), and other radiological modalities performed at the previous institution was investigated. NOFs/FCDs were categorized as follows based on Ritschl stage classification^[5,6]^: stage A, the lesion is small and oval without a sclerotic border and eccentrically located in the cortex near the epiphyseal plate (Fig. 2A); stage B, the lesion is lobulated, with a sclerotic margin and, in some cases, the cortex bulges without a periosteal reaction, with an increased size, and situated at a variable distance from the epiphysis (Fig. 2B); stage C, the lesion is characterized by increasing sclerosis, ordinally, from its diaphysis towards the epiphysis (Fig. 2C); and stage D, the lesion is completely homogeneously sclerotic (Fig. 2D). The radiological findings for NOFs/FCDs changed from stage A to B, C, and D according to age over several years. The relationships between the Ritschl stages and suspected diagnoses by primary physicians were compared.

### 2.4. Ethical considerations

This study was performed in accordance with the relevant guidelines and regulations and was approved by the institutional review board. As a retrospective chart review, consent for participation in this study was waived, and the waiver was approved by from the institutional review board.

## 3. Results

Overall, 108 patients (62 male and 46 female) were retrospectively included in this study. The median age at diagnosis was 11 (range, 5–32) years. The demographic data of patients are shown in Table 1. Regarding the initial finding, the lesion was incidentally discovered on plain radiography during an investigation for another clinical problem in 36 cases while the initial symptom was pain in 69 cases. The affected sites included the femur (67 cases), tibia (32 cases), fibula (7 cases), and humerus (1 case). Multiple lesions were detected in the distal femur and proximal tibia in 1 case, while femoral lesions were bilaterally distributed in 10 cases.

The primary physicians were affiliated with hospitals and clinics in 45 (41.6%) and 63 (58.3%) cases, respectively, with orthopedic surgery being the most common specialty (105 cases, 92.5%). The suspected diagnoses recorded by the previous physicians in the patient referral documents are shown in Fig. 1. In 40 patients (37%), NOF/FCD was initially suspected, and in 48 (44.4%) cases, the initial diagnosis was classified as “No description.” Benign and intermediate bone tumors, such as osteoid osteoma, fibrous dysplasia, solitary bone cyst, and giant cell tumor of the bone, were suspected in 15 cases. Malignant bone tumors were suspected in 5 cases: osteosarcomas in 4 cases and malignancy (unknown bone tumor) in 1.

**Figure 3.**
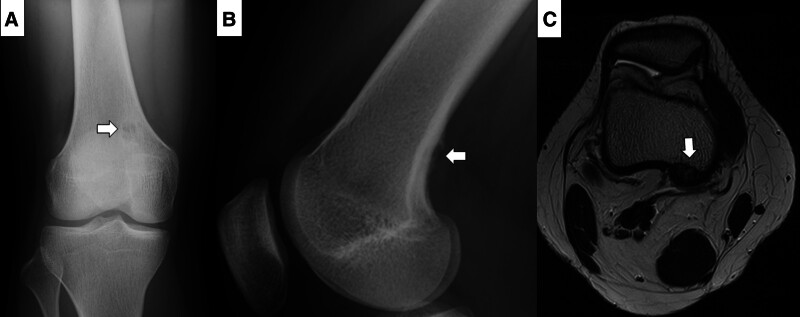
Fifteen-year-old female patient with a suspected osteosarcoma diagnosis made by the primary physician. The anteroposterior (A) and lateral (B) views of the plain radiograph show a small and oval osteolytic lesion with a sclerotic margin (Ritschl stage A). Subtle periosteal reactions are observed. (C) T2-weighted axial MRI confirming no soft-tissue extension from the posterior cortical portion.

**Figure 4.**
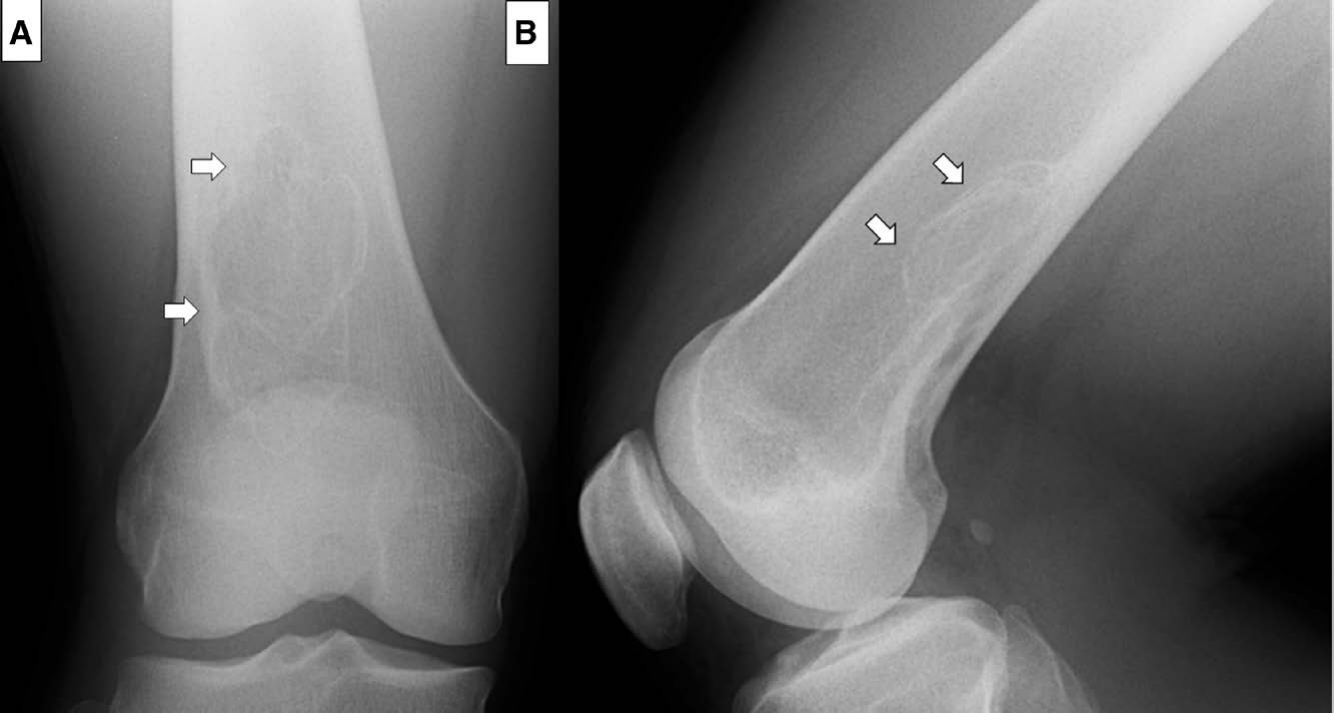
A 26-year-old male patient with a suspected malignant bone tumor diagnosis made by the primary physician. Anteroposterior (A) and lateral (B) views of the plain radiograph showing a large polycystic lesion with homogenous sclerosis (Ritschl stage D).

Referral documents for the 108 patients indicated that 39 patients underwent plain radiography, 56 had plain radiography and MRI, 5 had plain radiography and CT, and 6 had plain radiography, MRI, and CT. Additionally, 1 patient underwent MRI and another underwent both MRI and bone scintigraphy. Regarding suspected malignant bone tumors in 5 cases (Table 2), 3 of the 4 cases showed typical radiological findings of NOFs or FCDs, presenting with small marginal sclerosis (Cases 1, 2, and 3). The patient in Case 2 had previously undergone systemic chemotherapy for leukemia, and considering the late adverse effects related to systemic chemotherapy, the physician may have been concerned about the possibility of osteosarcoma. In Case 4, a 15-year-old female patient showed a subtle periosteal reaction with marginal sclerosis in the right distal femur (Fig. 3A and B), and FCD was confirmed on MRI, owing to the lack of soft-tissue extension (Fig. 3C). In Case 5 (26-year-old man), radiologically, the lesion exhibited large but homogeneous polycystic sclerosis with clear margins. The lesion was classified as Stage D (Fig. 4A and B).

**Table 2:** Patients suspected of having malignant bone tumors.

Case	Age/sex	Suspected diagnosis	Lesion	Symptom	Previous physician	Past history	Ritshl-stage
1	14M	Osteosarcoma	Tibia	Incidental discovery	Orthopedic surgeon	None	A
2	9M	Osteosarcoma	Femur	Pain	Orthopedic surgeon	Leukemia	A
3	10M	Osteosarcoma	Femur	Pain	Orthopedic surgeon	None	A
4	15F	Osteosarcoma	Femur	Pain	Orthopedic surgeon	None	A
5	26M	Malignant tumor	Femur	Incidental discovery	Orthopedic surgeon	None	D

The relationship between Ritschl stages and suspicious diagnoses was compared. In 64 patients with stage A, NOFs/FCDs were diagnosed in 38.1% (24) of the cases, and malignant bone tumors were suspected in 4.8% (3) cases (Table 3). In 30 patients with stage B, NOFs and FCDs were diagnosed in 36.7% (11) of the cases, and malignancy was suspected in 1 (9%). For stages C and D, 3 (30%) and 2 (40%) cases were diagnosed as NOFs/FCDs, whereas no malignancy was suspected in Stage C. Meanwhile, malignant bone tumor was suspected in 1 case with Stage D. The presumptive diagnoses for “No description” were stages A, B, C, and D in 46%, 43.3%, 40%, and 40% of the cases, respectively.

**Table 3:** Ritschl stages and suspected diagnoses made by previous primary physicians.

Stage	Number	NOF/FCD	Benign/intermediate	No description	Malignancy
A	63	24 (38.1%)	7 (11.1%)	29 (46%)	3 (4.8%)
B	30	11 (36.7%)	5 (16.7%)	13 (43.3%)	1 (9%)
C	10	3 (30%)	3 (30%)	4 (40%)	0 (0%)
D	5	2 (40%)	0 (0%)	2 (40%)	1 (20%)

## 4. Discussion

This study revealed that previous physicians initially diagnosed NOFs/FCDs in 37% (40 of 108) of cases. Twelve and 3 patients were diagnosed with benign and intermediate malignancies, respectively. Malignant bone tumors, including osteosarcoma, were suspected in 5 cases (4.6%). The remaining 48 patients (44.4%) were not diagnosed with anything specific, and their conditions were classified as “No description.”

NOFs/FCDs are classically categorized into different clinical entities^[7,8]^; however, these are the same in the histological process in bone.^[9,10]^ According to World Health Organization criteria, FCDs are confined to the cortex, whereas NOFs extend into the bone marrow, and the term FCD is no longer recommended.^[2]^ Therefore, the terms NOF/FCD were used synonymously in the present study.

The radiologic finding-based differential diagnosis of NOF/FCD includes fibrous dysplasia, solitary bone cyst, aneurysmal bone cyst, eosinophilic granuloma, chondromyxoid fibroma, bone infarction, desmoplastic fibroma, and benign fibrous histiocytoma.^[1]^ This study revealed that NOFs/FCDs were diagnosed in 37% (40 of 108) of the cases, and benign bone tumors were suspected in 12 cases. In the remaining 48 cases, the physicians failed to judge the benign nature of the lesions, and in 5 cases, the lesions were misdiagnosed as malignant bone tumors.

Osteosarcomas exhibit the radiological features of permeative or moth-eaten bone destruction with ill-defined borders, tumor-bone cloud-like opacities, aggressive periosteal reactions, and soft-tissue extension.^[11–13]^ They develop in the metaphyseal region of the long bones. However, in clinical practice, osteosarcomas mostly develop in patients of the same age group and anatomical sites as those with NOFs/FCDs. The majority of primary referring physicians were orthopedic surgeons (97.2%). IEven experienced orthopedic surgeons may find it challenging to distinguish between NOFs/FCDs and osteosarcomas. Studies report that primary physicians diagnose an average of only 0.7 cases of bone sarcoma over a median of 14 years.^[10]^ In this study, malignancy was suspected in 5 of the 108 cases, indicating a low rate of suspected malignant bone tumors.

The rate of suspected diagnoses classified as “No description” was unpredictably high (44%, 48 cases), potentially due to the distinct radiomorphological course of NOF/FCD stages. Stage A lesions are oval and small. Stage B lesions increase in size, suggesting locally aggressive bone tumors. Stage C lesions present with combined osteolytic and sclerotic radiological appearances. Stage D lesions are homogenously sclerotic. The transition of radiological appearance depending on lesion age is relatively peculiar in these lesions.^[14–16]^ Although these radiological alterations may lead to obscure presumptive diagnoses by primary physicians, especially in cases of large lesions of stages B and C, the rate of diagnosis of “No description” lesions was consistently high, at over 40%, from stages A to D. In this study, most primary referring physicians were not experienced specialists in bone tumors; therefore, the accurate diagnosis of bone tumors was less likely. However, physicians can acquire the ability to recognize the typical radiological features of NOFs/FCDs as possibly benign bone tumors, as these lesions are common around the knee region in children and young adults, and plain radiography are mostly diagnostic.

This study has some limitations. First, this was a retrospective study performed in a single cancer center in an urban area of Japan. Second, the number of patients with NOFs/FCDs was small. Third, referral documents were reviewed; however, they did not always reflect the actual knowledge and awareness of the primary physicians regarding NOF/FCD. Fourth, supplementary diagnostic findings of diagnostic radiologists were not considered in this study.

In conclusion, the radiological finding of NOFs/FCDs was diagnostic; nevertheless, lesions were diagnosed as “No description” at a high rate, indicating that primary physicians were not familiar with these lesions. NOFs/FCDs commonly develop in children and young adults. Continuous education regarding the typical radiological findings of NOFs/FCDs is necessary for primary physicians, mainly orthopedic surgeons.

## Author contributions

**Conceptualization:** Manabu Hoshi.

**Data curation:** Naoto Oebisu.

**Formal analysis:** Yoshitaka Ban, Naoki Takada.

**Methodology:** Masanari Aono.

**Software:** Tadashi Iwai.

**Supervision:** Masanari Aono.

**Writing – original draft:** Manabu Hoshi.

**Writing – review & editing:** Masanari Aono.
